# Impact of probiotic supplementation on exercise endurance among non-elite athletes: study protocol for a randomized, placebo-controlled, double-blind, clinical trial

**DOI:** 10.1186/s13063-022-06552-x

**Published:** 2022-07-27

**Authors:** Caitlin E. McDermott, Heather K. Vincent, Anne E. Mathews, Brunella Gonzalez Cautela, Mariana Sandoval, Annie Tremblay, Bobbi Langkamp-Henken

**Affiliations:** 1 Department of Food Science and Human Nutrition, University of Florida, 572 Newell Drive, PO Box 110370, Gainesville, FL 32611-0370 USA; 2 Department of Physical Medicine and Rehabilitation, College of Medicine, UF Health Sports Performance Center, University of Florida, 3450 Hull Road, PO Box 112730, Gainesville, FL USA; 3 UF Health Sports Performance Center, University of Florida, Gainesville, USA; 4 Lallemand Health Solutions, Montreal, QC Canada

**Keywords:** Non-elite athletes, Running, Ergogenic, Probiotics, Performance

## Abstract

**Background:**

Some probiotics appear to improve athletic performance, endurance, and recovery after intense exercise. Other formulations may provide performance-related benefits via immune and gastrointestinal functions in athletic individuals. However, few formulations have been studied for both types of effects among non-elite athletes. The primary objective of this study is to assess the ergogenic effects of a probiotic on high-intensity endurance running performance in non-elite runners. Secondary objectives include assessment of perceived exertion, blood chemistry, immune and stress biomarkers, cold and flu symptoms, and gastrointestinal health after the probiotic intervention.

**Methods:**

This 9-week randomized, placebo-controlled, double-blind, parallel trial will assess the ergogenic effects of a probiotic (5 billion colony-forming units/day, for 6 weeks) in healthy, non-elite runners (*N*=32; 18–45 years). Participants will be monitored via daily and weekly questionnaires during the 2-week pre-baseline, 6-week intervention, and 1-week washout. Questionnaires will inquire about activity, muscle soreness, gastrointestinal symptoms, cold and flu symptoms, stool form and frequency, and adverse events. During the pre-baseline visit, maximal oxygen uptake (V̇O_2_ max) is assessed to set appropriate individualized workload settings for the treadmill time-to-exhaustion endurance tests. These time-to-exhaustion endurance running tests will be completed at an intensity of 85% VO_2_max at baseline and final visits. During these tests, self-perceived exercise effort will be rated via the Borg Rating of Perceived Exertion scale and finger sticks assessing capillary blood glucose and lactate concentrations will be collected every 3 min. Additional questionnaires will assess diet and motivation to exercise. Body composition will be assessed using air displacement plethysmography at the baseline and final visits. Hypotheses will be tested using two-sided tests, and a linear model and with a type I error rate of *α*=0.05. Primary and secondary outcomes will be tested by comparing results between the intervention groups, adjusting for baseline values.

**Discussion:**

These results will build evidence documenting the role of probiotics on running endurance performance and physiological responses to exercise in non-elite athletes. Understanding the potential mechanisms of probiotic effects and how they mitigate the intestinal or immune discomforts caused by running could provide additional strategy means to help runners improve their performance.

**Trial registration number:**

ClinicalTrials.govNCT04588142. Posted on October 19, 2020.

Protocol version: July 2, 2021, version 1.2

## Introduction

### Background and rationale

Running is the most popular sport worldwide that confers numerous health benefits [1]. Intense exercise can trigger a stress reaction that negatively impacts the immune system, causes gastrointestinal discomfort, and compromises performance [2]. To mitigate stress-induced symptoms related to exercise and to improve endurance performance, athletes often turn to nutritional supplements, such as probiotics. The International Olympic Committee defined probiotics as live microorganisms that were “associated with a range of potential benefits to gut health, as well as modulation of immune function” [3]. In addition, the Food and Agriculture Organization of the United Nations and the WHO (FAO/WHO) defines probiotics as “live microorganisms that, when administered in adequate amounts, confer a health benefit on the host” [4]. Probiotics positively influence the immune system after intense exercise by decreasing both the intensity of respiratory symptoms and the risk of developing a respiratory infection, and by reducing gastrointestinal symptoms potentially through modulation of the gut microbiota, increased production of beneficial metabolites, and improvements in intestinal barrier function [5, 6]. Theoretically, through the modulation of training-induced symptoms, probiotics could enhance athletes’ ability to train at high intensities while avoiding training time interruptions, thereby improving performance [5]. Most studies investigating the effects of probiotics on gastrointestinal and immune symptoms associated with intense exercising yield conflicting results [7]. For example, Shing et al. administered 45 billion colony-forming units (CFU, a term used to describe the number of viable microorganisms in a probiotic supplement) of *Lactobacillus, Bifidobacterium*, and *Streptococcus* to male non-elite runners for 4 weeks; a “small to moderate” improvement on gastrointestinal permeability was detected, with no influence on inflammatory markers [8]. In contrast, a 4-week *Lactobacillus* (20 billion CFU) intervention in fatigued athletes restored the T-cell production of INF-γ (a pro-inflammatory cytokine mediating anti-viral and anti-bacterial immunity), to levels seen in healthy athletes [9]. Moreover, when administered for 14 weeks, a *Lactobacillus* probiotic (20 billion CFU) intervention maintained concentrations of salivary secretory immunoglobulin A (sIgA), a marker of mucosal immune function, and reduced the total number of upper respiratory infection days among elite athletes [10, 11].

Recent studies suggest that probiotics, namely *Lactobacillus*, could improve athletic performance. These ergogenic effects were detected in athletes with various sport specializations, including runners [7, 8, 12–15]. Huang et al. observed a dose-dependent improvement in time-to-exhaustion during treadmill testing in non-elite athletes (*N*=54, age 20–30 years) who received a *Lactobacillus* probiotic (30 billion CFU and 90 billion CFU) for 6 weeks [12, 13]. Similarly, time-to-exhaustion was improved in a running protocol after administering a probiotic formulation composed of *Lactobacillus*, *Bifidobacterium*, and *Streptococcus* to male non-elite runners (*N*=10, age 25–29 years) for 4 weeks [8]. Possible endurance-enhancing mechanisms include alteration of the gut microbiota which could impact muscle substrate use, increased glucose availability and enhanced antioxidant defense to combat exercise-induced oxidative stress [13]. In contrast, in elite athletes, there was no improvement in running endurance after a 14-week *Lactobacillus* intervention (*N*=50, age 20–25 years) compared to placebo [11].

Interpretation of the performance findings related to probiotics may be clouded by variations in training status of runners enrolled in the research. Some elite athletes who routinely train and perform at intensities near maximum V̇O_2_ max value may experience less ergogenic effect on time-to-exhaustion with probiotics compared to non-elite athletes [11, 16]. Probiotic administration in the non-elite athlete may produce greater effects on physiological responses to exercise such as endurance, perception of muscle effort, and energy metabolism (blood lactate and blood glucose). Non-elite runners may also benefit from probiotic-induced immunomodulatory and gastrointestinal effects that could modulate performance, but this has yet to be determined [17].

### Objectives

The primary objective of this study is to assess the ergogenic effects of a probiotic on high-intensity endurance running performance in non-elite runners. Secondary objectives include assessment of perceived exertion, blood chemistry, immune and stress biomarkers, cold and flu episodes, and gastrointestinal health after the probiotic intervention. We hypothesize that a 6-week probiotic intervention will increase time-to-exhaustion during running and reduce the number of gastrointestinal and cold/flu symptoms compared to the placebo.

### Trial design

This is a randomized, placebo-controlled, double-blind, parallel study design. Allocation was set in a 1:1 ratio (intervention: placebo) for this superiority trial.

## Methods: participants, interventions and outcomes

### Study setting

This trial is being conducted in a sports performance center of a tertiary care medical center, in accordance with the Good Clinical Practice-International Conference on Harmonization guidelines. All study procedures and documents have been approved by the University of Florida Institutional Review Board (18 December 2020, study # IRB202001882). This study is registered as a clinical trial on ClinicalTrials.gov, NCT04588142 (October 19, 2020). SPIRIT reporting guidelines were used in the development of this manuscript [18].

### Eligibility criteria for participants

Participants must meet the following inclusion criteria to be enrolled in the trial: (1) healthy adults between 18 and 45 years old; (2) running or cross-training 3–5 days per week at 45 min to 1.5 h per activity session, and willing to maintain this level of training throughout the study; (3) running ≥24 km per week; (4) willing to discontinue consumption of probiotic supplements and probiotic-fortified products throughout the study; (5) a V̇O_2_ max value in the 60–85th percentile (good-excellent health) range according to American College of Sports Medicine (ACSM) guidelines [19]; (6) willing and able to complete the informed consent from in English.

The exclusion criteria are as follows: (1) any physician-diagnosed diseases that would impact exercise performance or participation, including gastrointestinal disease, heart/cardiopulmonary disease, diabetes, thyroid disease, hypogonadism, hepatorenal disease, musculoskeletal disorder, neuromuscular/neurological disease, autoimmune disease, cancer, peptic ulcers or anemia; (2) professional or elite status; (3) currently smoking (including vaping); (4) positive results on COVID-19 test performed in the last 4 weeks and/or currently experiencing symptoms of COVID-19; (5) pregnancy before or during the study, planning to get pregnant or breastfeeding; (6) severe lactose intolerance and/or allergy to milk, soy, or yeast; and (7) used any antibiotic drug within 4 weeks of randomization. If antibiotic treatment is started while on the study, the participant will not be withdrawn; however, the participant will be asked to take the antibiotic and the study supplement 6 h apart.

### Recruitment

Recruitment for this study will begin in February 2021 and is estimated to finish in February 2022. Potential participants will be recruited via flyers, posters, email announcements, social media announcements, word-of-mouth, and sharing the opportunity at local running group meetings. Interested participants will complete a self-screening questionnaire and then study coordinators will contact the potential participant via phone or email.

### Informed consent

All participants will provide written informed consent after clear explanation of the trial by qualified, experienced members of the research team (study coordinators) prior to any trial-related procedures. Participants will be consenting to all trial procedures and potential future studies that will use their deidentified stool samples. All consenting procedures will be performed in a private area in the UF Health Sports Performance Center. The study coordinator will review the entire form and answer any questions from the potential participant.

### Probiotic intervention

The comparators were chosen to provide either active probiotic or inert placebo. The probiotic (5×10^9^ CFU/capsule) and corresponding placebo were provided by Lallemand Health Solutions Inc. This dosage was selected to determine probiotic efficacy in this athletic population. Placebo capsules contain the same excipients, namely ascorbic acid, magnesium stearate, and potato starch, without the probiotic. The probiotic and placebo capsules are sensorially identical (same smell, taste, and look) which allows for allocation concealment. Participants will receive all study capsules (probiotic or placebo) at the randomization visit and will be instructed to consume one capsule daily with a meal for 6 weeks. Criteria for discontinuing the intervention will be the occurrence of any clinical adverse event (AE) related to the intervention, laboratory abnormality, or other medical conditions (e.g., pregnancy) or situations such that continued participation in the study would not be in the best interest of the participant.

### Compliance with intervention

Study monitoring will be performed by the study sponsor through monthly data monitoring forms. Daily and weekly questionnaires will assess participant compliance with consumption of the study supplement. The daily and weekly questionnaires will be administered electronically via Qualtrics Survey Software (SAP Software Solutions). Study coordinators will monitor daily questionnaires for completion and a second reminder will be sent to participants who do not complete daily questionnaires by 6PM each day. Missed daily questionnaires will be interpreted as if no capsule was taken that day, and the participant will be instructed to consume two capsules the following day. Compliance will be calculated as the number of capsules consumed divided by the number the participant was expected to consume. Noncompliance will be considered as <80% consumed. Stool samples collected before and after the intervention will be used to assess the presence of the probiotic strain and confirm compliance.

### Randomization, sequence generation, concealment, and blinding

The randomization scheme using pre-defined blocks will be generated using a random number generator by laboratory personnel unrelated to the study. Four codes will be used for the placebo and probiotic to preserve blinding in case an adverse event requires unblinding. Allocation will be done in a 1:1 ratio using sealed, opaque envelopes that will also conceal the randomization sequence. Participants will be stratified by sex and randomized on-site by trained staff into two parallel arms (*n*=16/arm) to receive either the probiotic or placebo. Participants and study personnel on-site (Principal Investigator and co-Investigators, research coordinator, and assistants) and off-site personnel involved in the study (project manager, laboratory manager, and technicians) will remain blinded throughout the trial. Upon study completion, the study sponsor will reveal which codes are paired. However, all research personnel related to the study will remain fully blinded until all data analyses are complete. Once all analyses are complete, all study personnel will meet for unblinding.

### Primary and secondary outcomes

Primary and secondary outcomes are summarized in Table 1. The primary outcome measure is the difference in time-to-exhaustion during the endurance treadmill test (run at the HR corresponding to 85% of V̇O_2_ max) between the probiotic and placebo groups after a 6-week intervention. Changes will be adjusted for corresponding baseline time-to-exhaustion values. Longer time to exhaustion is a desired outcome for runners and offers a performance advantage in a competitive environment.

**Table 1:** Descriptions of the study outcome measures

Outcome	Description
Primary	
Time-to-exhaustion during treadmill endurance run	During an endurance treadmill run performed at the heart rate (HR) corresponding to 85% maximal rate of oxygen consumption (V̇O_2_max), the participant will run until voluntary time to exhaustion (reflected by meeting 2 out of 3 criteria: respiratory quotient ratio (VCO_2_/VO_2_) ≥ 1.1; HR reaches a minimum of 85% of age-predicted maximal HR (220 minus age); rating of 17 on the 6 to 20 point Borg Rating of Perceived Exertion scale (BRPE).
Secondary	
Blood chemistries	Capillary blood samples will be collected before, during, and after the treadmill endurance test to determine concentrations of glucose and lactate. These metabolites will be quantified using commercial, handheld colorimetric devices (ContourNext EZ glucose meter, LactatePlus lactate meter).
Body composition	This variable will be assessed using the scale (to assess weight) and BOD POD®, a body composition measuring device that measures fat mass and lean muscle mass. Coefficients of variation are 12%, and ICC are 0.9 [20].
Borg Rating of Perceived Exertion Scale (BRPE):	Exertion is a subjective estimate of exercising intensity. This self-reported instrument assesses effort and fatigue due to physical work [21]. Ratings range from 6=no muscle effort to 20=maximal muscle effort possible, cannot work any harder.
Bristol Stool Scale (BSFS):	Is a 7-point, self-rated scale of changes in stool form (hardness, shape, water content) that corresponds to intestinal transit time (*r*=−0.65) [22]. This tool is being used to monitor responses to the intervention and is supported for use in gastrointestinal research.
Daily Questionnaire:	Is a self-administered, online, daily questionnaire. The items include muscle soreness, consumption of supplements other than the study supplement, and compliance. There are also questions on cold/flu symptoms (used in previous trials) [23].
Gastrointestinal Severity Rating Scale (GSRS):	This assesses the severity of gastrointestinal symptoms. The GSRS scores 15 items evaluated in a 7-point Likert scale (1–*No discomfort* to 7–*Very severe discomfort*). The questionnaire is composed of a total score (sum of scores from all 15 items) and five subscales: abdominal pain (questions regarding abdominal pain, hunger pains, and nausea), constipation syndrome (questions regarding constipation, hard stools, feeling of incomplete evacuation), diarrhea syndrome (questions regarding diarrhea, loose stools, urgent need for defecation), indigestion syndrome (questions regarding rumbling, bloating, burping, gas) and reflux syndrome (questions regarding heartburn, acid regurgitation). Scores for each question are averaged to provide the sub-scale score. Reliability of the subscales is relatively good, with the Cronbach’s alpha ranging 0.61 to 0.83 [24].
Digestion-associated Quality of Life Questionnaire (DQLQ):	The impact of GI symptoms on quality of life among healthy adults is measured with this survey [25]. The DQLQ reflects the sum from 9 statements scored on a 10-point Likert scale (0 = *never* to 1 = *always*). This questionnaire has high convergent validity with GSRS-QOL scores r=0.54. Test-retest reliability between the DQLQ and GSRS were reported as ICC=0.89 [25].
Immune and Stress Biomarkers:	Salivary levels of secretory immunoglobulin A (sIgA), alpha-amylase, and cortisol are biomarkers of systemic immune function and stress. Biomarkers will be quantified using ELISA methods using commercial kits.
Covariates	
Short Healthy Eating Index (sHEI):	This instrument is designed to assess nutrient intake [26]. This is a self-report instrument designed to assess quality of diet relative to the American Dietary Guidelines. This validated instrument assesses 22 items to characterize dietary intake and yields a score between 0 and 100. The higher the score, the more the dietary quality aligns with the American Dietary Guidelines.
Fiber Screener:	This is a free-access tool created by NutritionQuest [27]. The range of possible results is between 15 g of fiber daily to 54 g of fiber daily. Higher grams of fiber consumed daily is associated with better general health. Validity was assessed using Spearman rank-order correlation coefficients between this short food screener and a full-length Food Frequency Questionnaire. Fruits and vegetable correlations were reported as *r* =0.71.
Behavioral Regulation in Exercise Questionnaire (BREQ-3):	This assesses the motivation for exercising. The questionnaire operationalizes motivation into six weighted scales: external regulation, introjected regulation, identified regulation, intrinsic regulation, integrated regulation and amotivation—which count with four items. Positive scores are associated to intrinsic motivation, which is better on the long term for exercising, while negative results are associated to extrinsic and lack of motivation. The questionnaire has demonstrated factorial reliability that was assessed using confirmatory factor analysis with LISREL 8.51 [28].
Stool Biochemistries	Probiotic strain recovery will be performed on stool samples before and after the intervention to support associations between outcomes and the probiotic. DNA will be extracted from homogenized stool samples using the ZymoBIOMICS 96 MagBead DNA kit (Zymo Research, cat# D4308). The absolute quantification is achieved using the CFX384 Touch Real-Time qPCR Detection System (Bio-Rad Laboratories) according to previously described methods [29] using specific forward and reverse primers.

The secondary outcomes, which will compare probiotic and placebo groups while adjusting for the corresponding baseline values, are as follows: (A) Borg Rating of Perceived Exertion (BRPE) [21] at peak lactate threshold during the endurance treadmill test (represents greater perceived tolerance to running workload); (B) exercise-induced changes in blood chemistry, specifically glucose concentrations and lactate threshold during the endurance treadmill test (reflects fuel use patterns during running); (C) biomarkers of stress (salivary cortisol and alpha-amylase) and biomarkers of immune activity (salivary secretory immunoglobulin A; sIgA); (D) cold/flu symptoms (severity, duration and number of episodes) as self-reported on the daily questionnaire [23]; (E) gastrointestinal function and discomfort as assessed by the weekly administration of the Gastrointestinal Symptoms Rating Scale (GSRS) [24] and (Digestion-associated Quality of Life Questionnaire (DQLQ) [25] and stool frequency and consistency (Bristol Stool Scale; BSFS) [22] as self-reported on the daily questionnaire; and (F) change in body composition (% fat, % fat-free mass, fat-free mass, fat mass, and body mass) from baseline between the probiotic and placebo groups.

### Sample size

The sample size of 32 (*n*=16 per arm), calculated using SigmaPlot v. 14.0, was based on a difference of 2.5 min between groups and standard deviation of 2.0 min in time to exhaustion during endurance treadmill testing at the final visit, as reported previously by Huang et al. [12, 13]. Using a two-sided sample *t*-test with a significance level of 0.05, it was calculated that a study with 24 participants, 12 per arm would have an 80% power to detect the assumed difference between the intervention groups. To adjust for an assumed dropout rate of 20% the total sample size would be 30. To evenly stratify the participants by sex in even blocks, two additional participants were added, bringing the total sample size to 32. This would provide for an effect size of 0.8, which is deemed a large effect.

### Overall study timeline and procedures

**Fig. 1:**
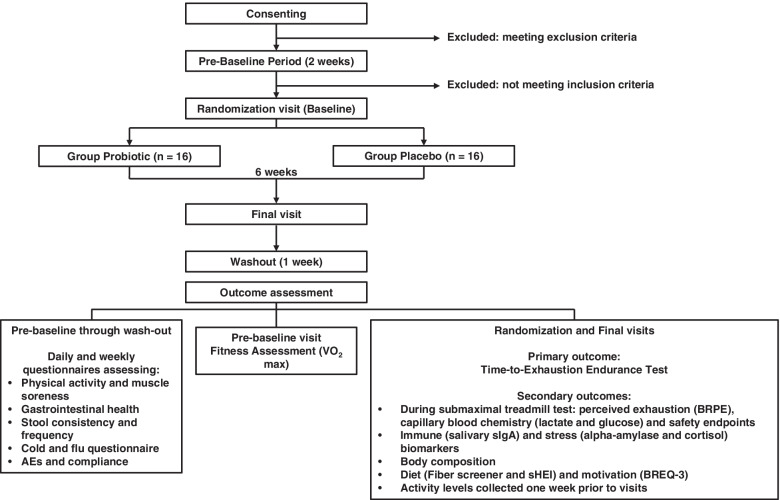
Study process diagram. AE, adverse events; BREQ-3, Behavioral Regulation in Exercise Questionnaire; BRPE, Borg Rating Perceived Exertion scale; sHEI, Short Healthy Eating Index; sIgA, secretory immunoglobulin A; VO_2_max, maximal rate of oxygen consumption

This study will consist of four visits in total, spanning over a period of 9 weeks (Fig. 1). Eligible participants will begin a 2-week pre-baseline period followed by the 6-week intervention, and 1-week post-intervention follow-up during which information on activity, muscle soreness, supplement intake, gastrointestinal and immune health, and adverse events will be collected via daily and weekly questionnaires. The following procedures will be conducted at each of the four visits (detailed in Table 2):

**Table 2:**
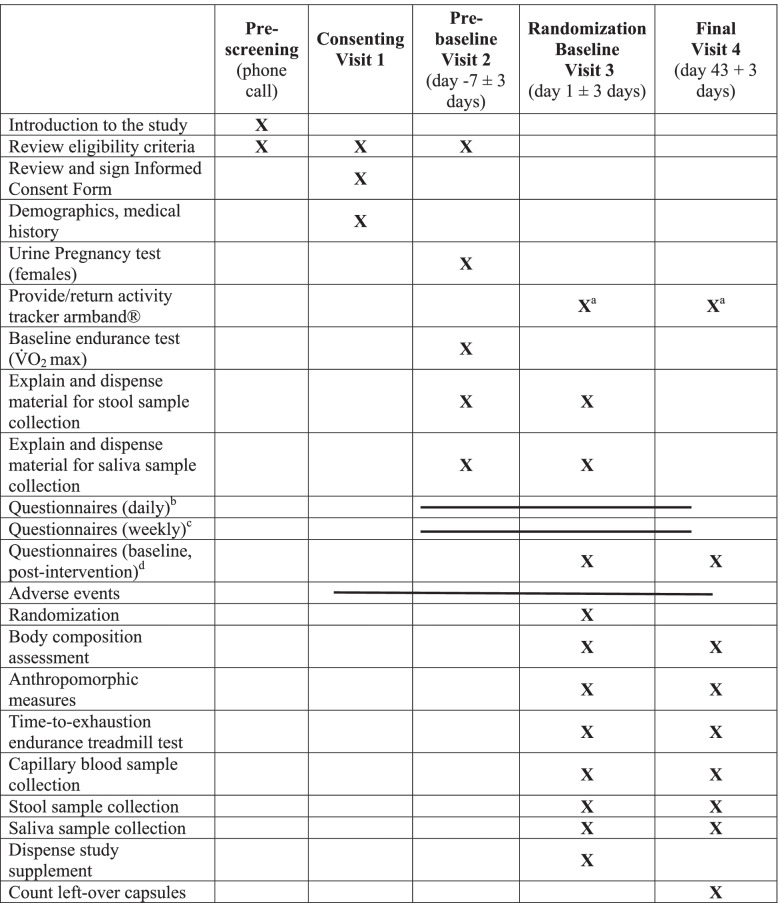
Schedule of activities per visit

^a^A week before the randomization and the final visits

^b^Questionnaires are administered daily, starting 14 days before the randomization visit until 7 days after the end of the intervention (muscle soreness, cold-flu symptoms, stool frequency-consistency)

^c^Questionnaires are administered weekly starting 2 weeks before the Randomization visit until 1 week after the end of the intervention (GSRS, BSFS, DQLQ)

^d^Questionnaires are administered at baseline and final visits (sHEI, Fiber Screener, BREQ-3)

#### Visit 1: Informed consent

Potential participants will be asked to read the informed consent form and meet with a study coordinator in a semi-private area to complete the informed consent process.

#### Visit 2: V̇O_2_ max and activity tracking

Occurring 1 week into the 2-week pre-baseline, visit 2 will comprise V̇O_2_ max testing, pregnancy screening for females, and pre-intervention physical activity tracking. Before proceeding with visit 2 activities, female participants will complete a urine pregnancy test; those who test positive for pregnancy will not complete the visit and be withdrawn from the study.

##### V̇O_2_ max testing

During this pre-baseline visit, participants will complete a modified Bruce V̇O_2_ max treadmill test protocol (Table 3) to determine their aerobic fitness level, following procedures outlined by the ACSM [19]. Blood pressures, subjective ratings of muscle effort, and ratings of angina and dyspnea will be captured in the final minute of each 3-min stage. Muscle effort will be self-rated using the 11-point Borg Rating of Perceived Exertion Scale (BRPE), and angina and dyspnea will be self-rated using 5-point scales, where 0 = no symptom and 4 = worst imaginable symptom. Breath-by-breath measurement of gas exchange will be captured using open-circuit spirometry (Viasys Sensormedics®) at rest, during exercise, and during recovery. During this test, the speed and grade of the treadmill will be increased every 3 min until the participant reaches a maximal effort. The test starts with a very light walking warm-up (stages 1 and 2) and progresses to faster walking and then to running until the following criteria are achieved to indicate a true maximal effort. A maximal effort requires achievement of nonprotein respiratory quotient (RQ; V̇CO_2_/V̇O_2_) ≥ 1.1; minimum heart rate (HR) at 85% of age-predicted maximal HR (220 minus age); rating of 17 on the BRPE; and a plateau of minute-by-minute V̇O_2_ values. After the test is complete, the corresponding HR at which 85% V̇O_2_ max occurred is identified and used to set the running speed in the time-to-exhaustion treadmill endurance tests in the next visits. If there are no abnormal cardiopulmonary responses during the V̇O_2_ max test, as defined by the ACSM guidelines, the participant will be cleared to continue in the study.

**Table 3:** Protocol for the modified Bruce V̇O_2_ max treadmill assessment to be completed at the pre-baseline visit

Stage	Time (min)	Speed (mph)	Grade (%)
Setup	−15:00–0:00		
20031	0:00–3:00	1.7	0
2	3:00–6:00	1.7	5
3	6:00–9:00	1.7	10
4	9:00–12:00	2.5	12
5	12:00–15:00	3.4	14
6	15:00–18:00	4.2	16
7	18:00–21:00	5	18
8	21:00–24:00	5.5	20
Recovery^a^	24:00–27:00	0	0

^a^For safety purposes participants will be asked to spend 15 min recovering during which heart rate and blood pressure will be measured but study measures will only be taken in the first 3 min

Any changes to physical activity levels could impact interpretation of the primary and secondary outcomes. Therefore, at the end of this visit, participants will receive an accelerometer activity tracker (SenseWear Armband Mini®) to record physical activity and sleeping patterns for 1 week (7 days) before randomization, with removal only while bathing/swimming. Depending on the speed, the percent error for step count in free living conditions for this device ranges from 0.9 to 13.7% [30].

#### Visit 3: Baseline treadmill endurance testing, biomarker sample collection, and randomization

**Fig. 2:**
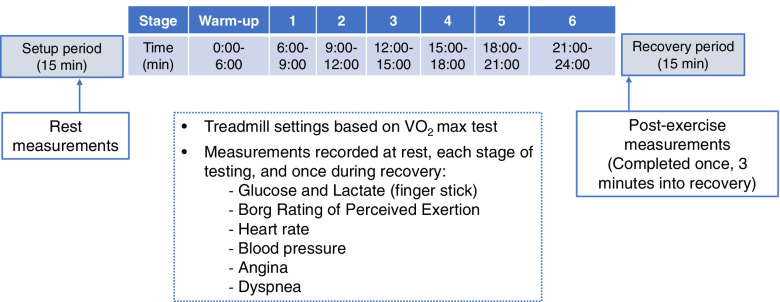
Treadmill endurance running test protocol for visits 3 (baseline) and 4 (final, 6 weeks post-intervention)

The morning of the first treadmill endurance test, participants will be assessed for several physiological features that could influence running performance or physiological responses to exercise independent from the probiotic intervention. These include stress and immune biomarkers, changes in body composition, motivation to exercise, dietary patterns, and fiber intake. Participants will collect saliva via passive drool in a commercial test kit (SalivaBio Collection Aid, Salimetrics; State College, PA) within 45 min of waking up to assess stress-related biomarkers including cortisol, alpha-amylase, and salivary sIgA (all described in Table 1). Participants will also provide stool samples in a nucleic acid preservation tube (Norgen Biotek, Corp; Ontario, Canada). Participants will then consume a standardized breakfast. Participants will be told to eat the same light breakfast consisting of lower-fat foods (e.g., yogurt with fruit, half a bagel or toast with peanut butter, oatmeal with fruit) before visits 3 and 4. Food intake will be recorded at visit 3. Participants will be reminded of this intake before visit 4. Participants will bring their saliva and stool samples to the testing site and return the activity tracker armband. In the lab, height, weight, and body mass index (BMI) will be measured. Body composition (e.g., proportions of fat-free and fat mass, body density) will be assessed using a medical grade scale and air displacement plethysmography via BOD POD® (COSMED USA, Concord, CA). Participants will complete a series of questionnaires and perform the time-to-exhaustion endurance treadmill test (Fig. 2). The short Healthy Eating Index (sHEI) is administered to assess dietary intake [26]. The Fiber Screener is a free-access tool created by NutritionQuest® [27]. The Behavioral Regulation in Exercise Questionnaire (BREQ-3) is administered to assess the motivation for exercising [28, 31]. These questionnaires will be used to track changes in patterns of food consumption, dietary fiber, and mental state regarding exercise, all of which may influence the primary and secondary outcome measures.

The treadmill endurance exercise test will be conducted at a level grade (Fig. 2). Participants will be asked to wear a HR monitor (Polar H10; Polar Inc; Dayton OH) throughout the test. The test consists of a 6-min warm-up period during which the speed of the treadmill will be increased until the participant achieves a HR of 85% of V̇O_2_ max and will continue at this speed until exhaustion or the participant asks to stop. Time-to-exhaustion is defined as meeting two out of three criteria: RQ ratio (VCO_2_/VO_2_) ≥ 1.1; HR reaches a minimum of 85% of age-predicted maximal HR (220 minus age); rating of 17 on the BRPE scale. Participants then remain seated for a recovery period where HR and blood pressure recover and are measured for safety before clearing participants to leave the testing area.

Throughout the test, HR, blood pressure, angina, dyspnea, and BRPE will be collected while finger prick blood samples will be used to measure glucose (Contour®Next EZ; blood glucose monitor; Parsippany, NJ) and lactate (Lactate Plus, Nova Biomedical; Waltham, MA) levels. These measurements will be collected at rest, 5 min into the treadmill test, then every 2–3 min until the participant reaches exhaustion, and once more 3 min after completion of the test.

After this visit, participants will begin their probiotic intervention and continue their daily and weekly questionnaires.

#### Visit 4: Post-intervention treadmill endurance testing

The procedures described for the endurance test, completion of questionnaires, salivary-stool biomarkers, and activity tracking with the armband described above for visit 3 will be repeated during this post-intervention visit. Participants will return any unused probiotic capsules for compliance calculation. Participants will complete daily and weekly questionnaires for an additional week after visit 4.

### Potential risks and discomforts

Potential risks may be associated with the probiotic or the treadmill testing***.*** Overall, probiotics do not pose a significant health risk and are generally regarded as safe. However, food allergy and gastrointestinal symptoms may occur in individuals who are allergic or intolerant to any of the ingredients in the probiotic. Treadmill testing (maximal or endurance testing) is associated with a minor risk of a fall or adverse cardiovascular complications. To minimize fall risks, spotters are present to support the participant should a fall occur, and a mirror is placed in front of the treadmill to help with spatial awareness. Although considered low risk, emergency equipment and a physician are available on site in the case of cardiac or vascular complications. If a participant experiences a complication believed to be related to the study, they will be withdrawn. Anticipated minor discomforts include finger tenderness during and after finger pricking from blood sampling. Lancet devices are used to minimize depth of puncture.

### Adverse events, seriousness, and reporting

An adverse event (AE) and serious AE will be determined and addressed per the International Council for Harmonisation of Technical Requirements for Pharmaceuticals for Human Use Guidelines for Good Clinical Practice [32].

All AEs and serious AEs will be captured on an adverse events report log. Serious AEs will be reported within 24 h of knowledge of the event to the sponsor and the IRB, commensurate with the determination of possible relation to the intervention. A written report will be submitted to the sponsor and IRB within 7 days of the initial reporting. Changes in the severity of an AE will be documented to allow an assessment of the duration of the event at each level of severity to be performed. AEs characterized as intermittent will require documentation of onset and duration of each episode. Study participants will be followed for 1 week after the final study supplement is consumed. If an AE occurs, which in the judgment of the study sponsor and investigators was directly caused by the study supplement or protocol activities, the study sponsor will pay for medical care as defined in the informed consent form.

### Confidentiality

Each participant will be given a pre-randomization number upon signature of the informed consent and will be assigned a randomization number upon randomization. These codes will be used to identify the participants throughout the study and during data collection and analyses. Source data and data generated during the visits will be collected using paper-based case report forms. All documentation, data, and all other information generated will be held in strict confidence in password-protected, encrypted computers. Data collected during the trial will be deidentified upon closure of the study. No personal information concerning the study data or participants will be released to any unauthorized third party. Once the study is completed and deidentifying information has been removed from study records, the sponsor will store the deidentified stool samples for use in future research.

### Analyses of biological samples

Saliva and stool samples will be frozen at −80 °C upon receipt. Stool samples will be shipped to the sponsor for analyses. DNA will be extracted from the stool samples for qPCR. Saliva samples will be used to measure salivary sIgA, cortisol, and alpha-amylase levels by enzyme-linked immunosorbent assay (ELISA) methods using commercial kits (detailed in Table 1).

### Statistical methods

The primary outcome will be tested using a linear model to determine if the adjusted mean response value differs between groups adjusting for baseline values. For secondary outcomes that measure the statistical difference in response between intervention groups (blood chemistries, perceived exertion, and salivary measures), a linear model will be used to determine if the mean response value differs between groups, adjusting for baseline values. Where relevant, differences between groups will be adjusted for sex as well as the corresponding baseline. For secondary outcomes that are only measured during the intervention period, the response between intervention groups (number of days sick, duration, and severity) will be tested by using a two-sample *t*-test. All analyses mentioned above will be performed with the intent-to-treat and per protocol populations. The per protocol population is defined as compliant participants who took at least 80% of the study supplement during the 6-week intervention period.

All hypotheses will be tested using two-sided tests with a type I error rate of *α* = 0.05 using the SAS v9.4, JMP Pro v14 (SAS Institute Inc., Cary, NC, USA), or SigmaPlot v14 (Systat Software, Inc.) software. For the continuous response data with models that require the normality of residuals and a homogeneous variance, the residuals will be assessed using plots of the standardized residuals in four ways: versus the predicted values, in a histogram, in a Q-Q plot versus the expected value under the assumed distribution, and in a boxplot. Should there be a failure of model assumptions then transformations will be applied, or non-parametric tests will be used where appropriate. The most common transformations used adjust for a skewness and heterogeneity in variance are the natural log, log base 10, or a square root transformation. If data are transformed for analysis, the least square means will be back-transformed for reporting and the standard errors of the back-transformed means will be estimated using the Delta method ^29^.

No imputation will be performed for missing variables where there is only a single outcome measured. Variables assessed daily but analyzed weekly (such as frequency and consistency of stool samples) will be averaged in the case of missing data. The variables assessed daily will be compared as the daily average per week.

### Frequency of auditing trial activity

Study case report forms will be reviewed on a weekly basis by a minimum of two investigators for quality control. Considering that this trial is low risk and of short duration, no interim analysis is planned. Monthly auditing checks will be conducted by the study sponsor to review adverse events, protocol deviations, supplement management, and recruitment numbers. Additionally, monitoring visits will be conducted by the study sponsor every 6 months.

### Communication of protocol amendments to relevant parties

All proposed protocol changes will be reviewed by study personnel and must be agreed upon between the study site and study sponsor before seeking IRB approval of the amendment.

### Dissemination plans

Study findings will be disseminated at scientific conferences and by publication in the trial registry or in a peer-reviewed journal by the members of the study team.

## Conclusion

The study protocol described here was designed to gather insight into the ergogenic effects of a probiotic in non-elite athletes. Additionally, the current study will assess the effects of the probiotic on gastrointestinal and immune health in non-elite runners. These results will help build the evidence base necessary for documenting the role of probiotics in non-elite athletes, with the ultimate goal of identifying additional options to help runners improve their performance and possibly overcome some of the intestinal or immune discomforts caused by running.

## Trial status

The trial is currently enrolling participants and in the data collection phase. Protocol version 1.2, July 2, 2021, is being used. Recruitment began in February 2021 and is estimated to be completed in February 2022.

## Data Availability

After publications of the results, deidentified data will be shared with qualified researchers and scientists upon reasonable request to the sponsor including a detailed proposal of the intended use of the data as per the Lallemand Health Solutions Inc. Policy on Clinical Trial Transparency and Data Sharing (available upon request).

## Abbreviations

ACSM: American College of Sports Medicine
BREQ-3: Behavioral Regulation in Exercise Questionnaire
BMI: Body mass index
BREQ3: Behavioral Regulation in Exercise Questionnaire
BRPE: Borg Rating of Perceived Exertion
BSFS: Bristol Stool Scale
CFU: Colony-forming units
DQLQ: Digestion-associated Quality of Life Questionnaire
ELISA: Enzyme-linked immunosorbent assay
GSRS: Gastrointestinal Symptoms Rating Scale
HR: Heart rate
RQ: Respiratory quotient
sHEI: Short Healthy Eating Index
sIgA: Secretory immunoglobulin A
VCO2/VO2: Carbon dioxide production/oxygen consumption
V̇O_2_ max: Maximal rate of oxygen consumption
